# The Effect of VoorZorg, the Dutch Nurse-Family Partnership, on Child Maltreatment and Development: A Randomized Controlled Trial

**DOI:** 10.1371/journal.pone.0120182

**Published:** 2015-04-01

**Authors:** Jamila Mejdoubi, Silvia C. C. M. van den Heijkant, Frank J. M. van Leerdam, Martijn W. Heymans, Alfons Crijnen, Remy A. Hirasing

**Affiliations:** 1 EMGO+ Institute for Health and Care Research, VU University Medical Center, Department of Public and Occupational Health, Amsterdam, the Netherlands; 2 Dutch Health Care Inspectorate, Amsterdam, the Netherlands; 3 Department of Epidemiology and Biostatistics, VU University Medical Center, Amsterdam, the Netherlands; 4 De Waag, Center for Forensic Services, Amsterdam, the Netherlands; University of Alabama at Birmingham, UNITED STATES

## Abstract

**Background:**

Child maltreatment is a great public health concern that has long-term mental and physical health consequences and can result in death. We studied the effect of a nurse home visiting program on child maltreatment among young disadvantaged families in the Netherlands. This study is the first to investigate the effects of this program outside of the United States.

**Methods:**

We conducted a single blind, parallel-group, randomized controlled trial that compared usual care with the nurse home visitation program, which began during pregnancy and continued until the children’s second birthdays, in 460 disadvantaged women who were pregnant for the first time and <26 years of age. The primary outcome was the existence of a report about the child from a child protecting services agency (CPS reports). Secondary outcome measures included home environment and child behavior.

**Results:**

Two hundred twenty-three participants were assigned to the control group, and 237 were assigned to the intervention group. Three years after birth, 19% of the children in the control group had a CPS report. The 11 percent of children in the intervention group with CPS files was significantly lower (relative risk 0.91, p-value 0.04). At 24 months, the intervention group scored significantly better on the IT-HOME. At 24 months after birth, the children in the intervention group exhibited a significant improvement in internalizing behavior (relative risk 0.56, p-value 0.04) but no evidence of a difference from the control group in externalizing behavior (relative risk 0.71, p-value 0.12).

**Conclusion:**

The number of CPS reports for the intervention group was significantly lower than that of the control group. Additionally, the long-term home environments were improved and internalizing behaviors of the children were lower in the intervention group.

**Trial Registration:**

Dutch Trial Register NTR854

## Background

Child maltreatment is a major public-health problem that is associated with grave physical and mental health and developmental consequences. Child maltreatment is associated with physical injury, growth retardation, obesity, anxiety, depression, posttraumatic-stress disorder, and long-term deficits in educational achievement.[1,2] Children die every year due to child maltreatment, although the actual number of deaths is unclear.[3] In adolescence, those who suffered from child maltreatment are more likely to be addicted to drugs and alcohol and to engage in risky behavior, such as juvenile delinquency, risky sexual behavior and dating violence. In adulthood, those who suffered from childhood maltreatment are more likely to have psychosocial problems and chronic diseases.[2,4] Furthermore, when these children become parents, they are at risk to abuse their own children.[1] The societal consequences of child maltreatment are also enormous not only in terms of direct costs but also in terms of greater use of community resources and lower levels of occupational functioning and employment. [5] The mortality and morbidity associated with child maltreatment are assumed to be potentially preventable.[1]

Article 19 of the United Nations Convention on the Rights of the Child states that, *“Governments must do all they can to ensure that children are protected from all forms of violence*, *abuse*, *neglect and mistreatment by their parents or anyone else who looks after them*.*”* However, despite the negative effects of maltreatment on child development, most programs aim only at secondary prevention rather than primary prevention of child maltreatment. The Nurse-Family Partnership (NFP) is an evidence-based program for the primary prevention of child maltreatment that was developed by Olds et al. [6] The NFP is a nurse home visitation program in which high-risk pregnant women receive well-structured home visits during pregnancy until the child’s second birthday. The effectiveness of NFP in reducing child maltreatment has been evaluated in three randomized controlled trials (RCT) that were all conducted in the United States only. [7–9] The Elmira trial showed that at ages 2 and 15 of the child the numbers of reports of child maltreatment to Child Protective Services (CPS) appeared to be significantly reduced among the NFP families.[8] Despite the effectiveness of NFP, this program has not been replicated in independent studies to date. In this study, we describe the effectiveness of VoorZorg, which is the Dutch adaption of the NFP, on the primary prevention of child maltreatment. The term VoorZorg has the connotations of both precaution as well as care at an early stage. To the best of our knowledge, this report describes the first RCT of the effectiveness of NFP outside the US.

## Methods

### Ethics Statement

Detailed descriptions of the design have been published elsewhere.[10, 11] In short, this is a single blind, parallel-group, randomized controlled trial (RCT) of VoorZorg. The (original) protocol for this trial and supporting CONSORT checklist are available as supporting information; see S1 Study Protocol, S1 Original Study Protocol and S1 CONSORT Checklist. The NFP was translated and culturally adapted into VoorZorg.[12] The Medical Ethical Committee of the VU University Medical Center approved the study in December 2006. Patient recruitment began in January 2007 and follow-up began in March 2007. The participants signed an informed consent statement. We did exclusively obtain written informed consent from the participants (pregnant women), who also gave us permission to obtain CPS data on their children. This procedure was approved by the ethics committee of our university. We recorded all informed consent forms both on the computer by scanning all forms as in an archive.

This trial is registered with the Dutch Trial Register (number NTR854) in 2006 before the first participant was included. This Register is the primary register in the Netherlands. Afterwards we also registered in the Current Controlled Trial. The authors confirm that all ongoing and related trials for this intervention are registered.

### Study design and participants

From January 2007 to April 2009, 460 participants were recruited for an RCT through a two-stage selection procedure. In the first stage, general practitioners, midwives, gynecologists and others actively recruited women in 20 municipalities in the Netherlands using the following five criteria: < 26 years of age, low educational level (pre-vocational secondary education), first time pregnancy, maximum 28 weeks of gestation, and some understanding of the Dutch language. These criteria are based on a literature study and were similar as the criteria in the NFP. [13,14] Recruitment occurred in formal settings, such as primary and secondary health care practices, and in informal settings, such as community centers. Women who met all five criteria were assigned to the second stage of the selection procedure in which VoorZorg nurses interviewed women to assess whether they had at least one of nine additional risk factors (i.e., being single, a history or present situation of domestic violence, psychosocial symptoms, unwanted pregnancy, financial problems, housing difficulties, no employment and/or education, or alcohol and/or drug abuse).

### Randomization and masking

All eligible women were stratified by region and ethnicity and randomized into a control or intervention group by an independent researcher of the VU University Medical Center. Ethnicity was based on participants’ self-reports. A participant was classified as a certain ethnicity if at least one of her biological parents was born in a country outside the Netherlands. Randomization was blind and accomplished through the use of a computer-generated list of random numbers (0, 1) created with the SPSS 14.0 software. Participants were assigned to the intervention or control group in a 1:1 ratio. The researcher then informed the VoorZorg nurses about the allocation. The interviewers were blinded to allocation, but it was not feasible to mask the participants or the care-givers to the allocations.

### Intervention

The women in the control group received the usual care. [11] The women in the intervention group received the usual care plus the VoorZorg program. The VoorZorg program consisted of approximately 10 home visits during pregnancy, 20 during the first, and 20 during the second year of the life of the child by trained and experienced VoorZorg nurses. Nurses were trained prior to implementing the intervention, received regular supervision in their organizations, and received one-day training sessions at the national level twice a year. Training was performed by the Netherlands Youth Institute. Training consisted among others of role plays to practice real-life situations. In addition, nurses reviewed each other by attendance at home visits alongside the nurse home visitors. During each home visit, topics in 6 different domains that were relevant to the stage of pregnancy and the development of the child were addressed. These visits are described in well-structured manuals in which the goals, procedures and content of each visit are elaborated. [15–17] The manuals are translated from the Nurse-Family Partnership. VoorZorg nurses offered health education and aimed to teach women parenting skills, to enhance their self-efficacy to reduce risk factors of child maltreatment and to improve the utilization of social and community resources. In addition to the home visits, VoorZorg nurses also communicated with the participants via text messaging, telephone and social media. It is essential to the VoorZorg program that the nurses establish an enduring and trusting relationship with the participants.

### Study outcomes

In the RCT on the effectiveness of VoorZorg the following outcomes were measured:

- Maternal cigarette smoking at 16–28 weeks and 32 weeks of pregnancy and two months after birth as well as maternal smoking near the child [18];
- Adverse pregnancy outcomes, birth weight and gestational age[18];
- Breast feeding [13]
- Intimate Partner Violence [12]
- Child development at six months, 18 months and 24 months of age, measured with, among others, the Home Observation for Measurement of the Environment, and the Child Behavior Checklist [26] [27];
- Child abuse reports.

This manuscript specifically addresses child abuse reports (primary outcome measure) and child development (secondary outcome measure).

### Primary outcome measure

In the Netherlands, both professionals and citizens, such as family members, can report any case of suspected child maltreatment to a Dutch CPS agency (Advies en Meldpunt Kindermishandeling in Dutch). [19] According to the CPS, 93% of reports to the CPS are valid cases of child maltreatment.[20] Child maltreatment is defined as: physical abuse, physical neglect, emotional/ psychological abuse, emotional/psychological neglect, or sexual abuse. The primary outcome was whether the child was reported to CPS within three-and-a-half years after randomization (pregnancy and first three years of life of the child). In the Netherlands there are approximately 29 CPS regions. We contacted ten CPS regions in which VoorZorg was carried out. The eight CPS agencies that were willing to cooperate were sent a list with the names and the most recent addresses of the children living in their region and were asked to indicate whether CPS reports related to those children had been filed. We could not send the names of all children to all CPS regions, because of privacy reasons. All participants gave permission (written) to obtain CPS data on their children.

### Secondary outcome measures

The secondary outcomes were assessed with questionnaires that were administered by trained female interviewers in the participants’ homes. To decrease the participants’ urges to provide socially desirable answers and for safety reasons, the interviewers requested that the interviews with the participants be conducted in private.

The Home Observation Measurement of the Environment (IT-HOME) was used at 6, 18 and 24 months of age to assess the environment of the child. The psychometric properties of this tool are: the inter-observer agreement is 0.80, and the internal consistency is 0.80. [21] The total IT-HOME consists of 45 items that are scored as “yes” or “no”, and the total scores is calculated as the sum of all positive scores. Higher total scores indicate more positive environments. Additionally, at 24 months after birth, the interviewers administered the Child Behavior Checklist 1.5–5 years (CBCL/1.5–5) to the mothers to assess the children’s behavioral problems. [22] The psychometric properties of this tool are as follows: the inter-observer agreement is > 0.50, and the internal consistency is between 0.78 and 0.92.[23] The “internalizing behavior” and “externalizing behavior” subscales were used, and children were considered to exhibit internalizing or externalizing behaviors if they scored ≥ the 90th percentile.

At baseline we assessed demographic characteristics and the following risk factors: being single, a history or present situation of domestic violence, psychosocial symptoms, unwanted pregnancy, financial problems, housing difficulties, no employment and/or education, or alcohol and/or drug abuse.

### Power Calculation

The main outcome of the entire study to calculate power was a reduction of four cigarettes smoked per day during pregnancy in the intervention group. [24] This was based on the results of the NFP study. [15] To observe an average improvement or a decrease in smoking by four cigarettes a day with a standard deviation of eight cigarettes, a power of 80% and an alpha of 5% were used. This resulted in a sample size of 57. Given that 25% of all women smoke at the start of pregnancy in the Netherlands, 228 participants in the control group and 228 participants in the intervention group were needed to detect a statistically significant effect. Positive effects of VoorZorg on smoking were observed in an earlier study. [18]

### Statistics

The data were analyzed with the SPSS 20.0 statistical package for Windows. The outcomes of the CPS reports and CBCL/1.5–5 were analyzed with Poisson regression models to assess the differences between the control and intervention groups. Relative risks (RR), absolute risk differences (ARD) and their corresponding Confidence-Intervals were calculated using a Poisson log-linear model according to Zou.[25] For the primary outcome, we conducted moderation analyses to test for differences between subgroups (gender of the child and ethnicity).[26] For the missing CBCL/1.5–5 data at 24 months after birth (49%), we applied multiple imputation (MI) analyses and validated these analyses with sensitivity analyses with the IBM SPSS statistics 20 program and generated 50 imputed datasets as recommended.[27,28] The total IT-HOME scores were first analyzed with multiple linear regression to measure group differences and subsequently analyzed with mixed model analyses to measure the longitudinal relationship between the VoorZorg intervention and the IT-HOME score over the three measurements. Because mixed model analyses resulted in a higher power we did not apply MI analyses for the IT-HOME score. Differences were considered significant when the p-values were <0.05 (2-sided). All analyses were adjusted for possible confounders and effect modifiers (region, age, ethnicity, gender of the child, age mother, weeks of gestation and birth weight). We conducted attrition analysis to evaluate differences on baseline characteristics between participants who remained in the study versus those who did not.

## Results

### Baseline characteristics

**Fig 1:**
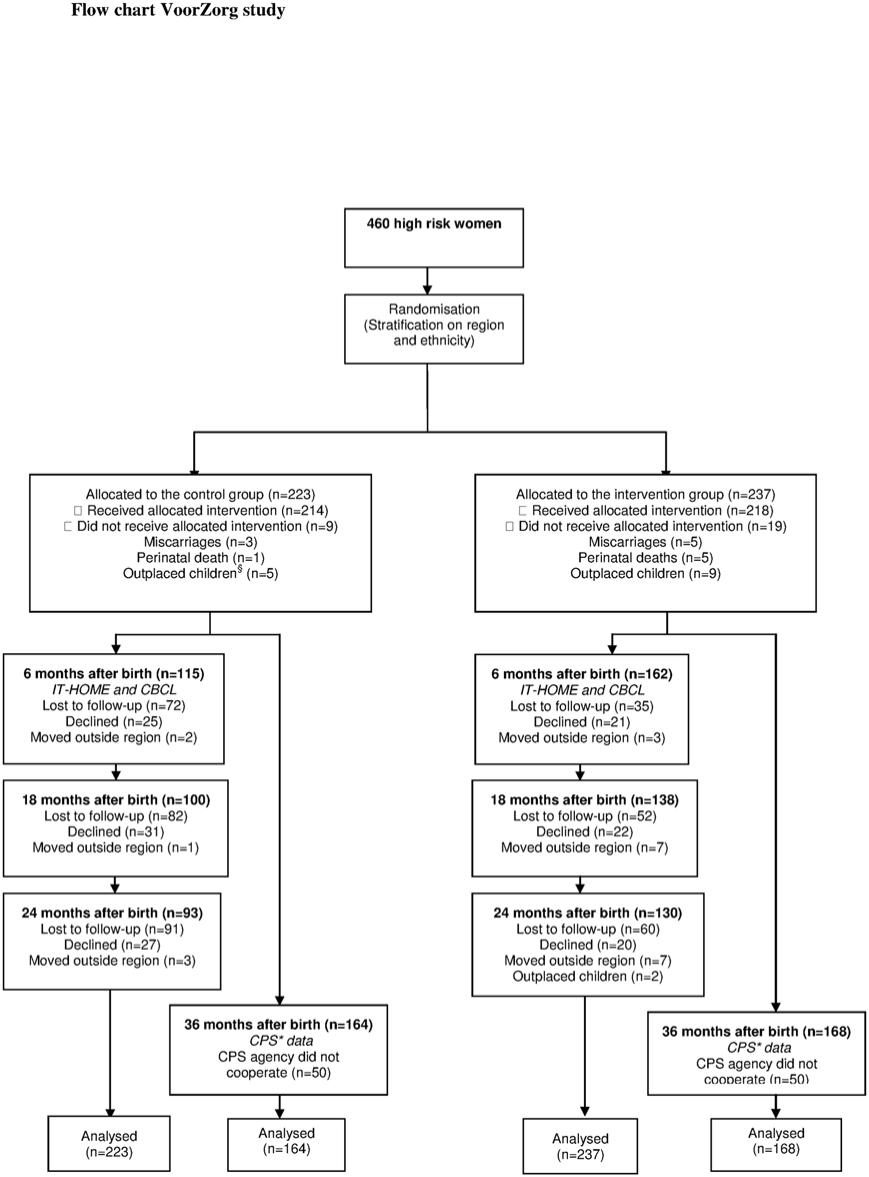
Flow chart VoorZorg study. ^§^ When the development of the child is in danger the juvenile court can impose an outplacement of the child. *CPS stands for Child Protective Services; in the Netherlands this organization is called AMK.

Of the 460 participants, 223 women were assigned to the control group and 237 were assigned to the intervention group. We contacted only CPS regions in the Netherlands where participants lived. Eight of these ten CPS regions (both urban and rural) agreed to participate in this study. All children in these eight regions (164 children in the control and 168 in the intervention group) were assessed whether they had a CPS report (Fig. 1). There were no significant differences in the characteristics of the CPS regions that cooperated in this study and those that did not. In Fig. 1 we have described the number of women that participated in each measurement. At baseline, no significant differences in demographic characteristics were observed between the control and intervention groups (table 1). The prevalence of risk factors at baseline was also similar across groups. As table 2 shows, attrition analysis showed no significant differences in baseline characteristics between participants lost to follow-up and participants who remained in the study.

**Table 1:** Baseline characteristics of participants in control (C) and intervention group (I).

	N		C	I
	C	I		
**Age, years mean(sd)**	214	233	19.1(2.3)	19.4(2.6)
**Weeks of gestation mean(sd)**	170	208	19.8(5.7)	19.5(6.0)
**Region**	223	237		
**Urban**			147(66)	158(67)
**Rural**			76(34)	79(33)
**Ethnicity**	223	237		
**Dutch**			110(49)	115(49)
**Turkish/Moroccan**			13(6)	13(6)
**Surinamese/Antillean**			58(26)	64(27)
**Other**			42(19)	45(19)
**Education level**	158	189		
**Primary school**			7(4)	11(6)
**Pre-vocational secondary education**			149 (94)	177(94)
**Higher education**			2(1)	1(1)
**Married/living together**	162	194	36(22)	46(24)
**Not having a boyfriend**	156	187	49(31)	70(37)
**Living with boyfriend**	161	194	40(25)	58(30)
**Lifetime prevalence of IPV** ^**1**^	160	199	84(53)	103(52)

*Note*. Numbers are n (%) unless described otherwise. N = Number for whom data are available

^1^IPV = Intimate Partner Violence.

**Table 2:** Comparisons between participants who completed the questionnaires and who were lost to follow-up on baseline characteristics for Control (C) and Intervention group (I).

	C			I		
	Completers	Non-completers	p-value	Completers	Non-completers	p-value
**Age, years mean(sd)**	19.4(2.4)	18.9(2.2)	0.11	19.4(2.7)	19.3(2.4)	0.66
**Weeks of gestation mean(sd)**	19.6(5.4)	20.0(6.1)	0.58	19.5(6.0)	19.5(6.1)	0.96
**Region**						
**Urban**	57(61)	90(69)	0.25	89(69)	69(64)	0.41
**Rural**	36(39)	40(31)		40(31)	39(36)	
**Ethnicity**						
**Dutch**	51(55)	59(45)	0.30	68(53)	47(44)	0.24
**Turkish/Moroccan**	4(4)	9(7)		4(3)	9(8)	
**Surinamese/Antillean**	25(27)	33(25)		34(26)	30(28)	
**Other**	13(14)	29(22)		23(18)	22(20)	
**Education level**						
**Primary school**	3(4)	4(5)	0.72	6(5)	5(7)	0.75
**Pre-vocational secondary education**	76(96)	73(94)		112(95)	65(92)	
**Higher education**	1(1)	1(1)		0	1(1)	
**Married/living together**	24(25)	15(19)	0.96	30(25)	16(22)	0.69
**Not having a boyfriend**	23(29)	26(34)	0.49	44(38)	26(36)	0.89
**Living with boyfriend**	24(29)	16(20)	0.20	36(30)	22(30)	0.96
**Lifetime prevalence of IPV** ^**1**^	46(55)	38(49)	0.44	64(53)	39(49)	0.58

*Note*. Numbers are n (%) unless described otherwise.

^1^IPV = Intimate Partner Violence

### Intervention delivery

Participants were included at 20 ± 6 (mean ± SD) weeks of pregnancy. They received on average 9 ± 4 (mean ± SD) home visits during pregnancy.

### Primary outcome

From pregnancy to three years after birth, 31 of 164 (19%) of the children in the control group had a CPS report, and in the intervention group18 of 168 (11%), which was significantly lower (RR for VoorZorg vs. Usual care, 0.58; 95% CI (0.28 to 0.96); ARD 0.08) (Table 3). Subgroup analyses stratified by the gender or ethnicity of the child revealed no significant differences in the primary outcome (data not shown).

**Table 3:** CPS reports, home environment and child behavior between ages 6 and 36 months in control (C) and intervention group (I).

		N		Mean (SD)/n(%)		RR/MD (95% CI)
		C	I	C	I	
**6 months**	**IT-HOME**	115	162	33.0(6.0)	33.4(6.9)	0.40(-2.75 to 2.04)
**18 months**	**IT-HOME**	100	138	36.8(6.1)	36.0(6.0)	0.80(-1.30 to 2.91)
**24 months**	**IT-HOME**	93	130	36.4(5.9)	38.3(4.8)	1.98(0.16 to 3.80)*
	**Internalizing**	93^1^	130^1^	69(31%)	40(17%)	0.56(0.24 to 0.94)*
	**Externalizing**	93^1^	130^1^	78(35%)	59(25%)	0.71(0.34 to 1.09)
**36 months**	**CPS reports**	164	168	31(19%)	18(11%)	0.58(0.28 to 0.96)*

*Note*. No confounders were found significant. N = Number for whom data are available; SD = Standard deviation; RR = Relative Risk, MD = mean difference; CI = Confidence Interval

^1^ The remaining participants had their data imputed

*p<0.05

### Secondary outcomes

Table 3 illustrates the results from the secondary outcomes. From 6 to 18 months after birth, the total IT-HOME scores increased in both groups; in the control group from 33.0±6.0 to 36.8±6.1, and the intervention group from 33.4±6.9 to 36.0±6.0. However, the difference between groups was not statistically significant. At 24 months after birth, the total IT-HOME score in the intervention group was significantly higher than in the control group (36.4±5.9 for the control group and 38.3±4.8 for the intervention group). Mixed model analyses (corrected for the age of the mother, ethnicity and the number of risk factors) revealed no significant differences between the groups over time in total IT-HOME scores (mean difference: 1.12; 95% CI: -0.59 to 2.83).

The prevalence of children with internalizing behavior according to the CBCL at 24 months (C: 31% vs. I: 17%) was significantly lower in the intervention group than in the control group (RR for VoorZorg vs. Usual care group: 0.56; 95% CI 0.24 to 0.94; ARD: 0.14). The prevalence of children with externalizing behavior (C: 35% vs. I: 25%) was not significantly different across groups (RR for VoorZorg vs. Usual care group: 0.71; 95% CI 0.34 to 1.09; ARD: 0.10).

## Discussion

Despite the negative impact of child maltreatment, the Nurse-Family Partnership (NFP) by Olds et al. is the first evidence-based program for the primary prevention of child maltreatment.[8] The NFP is a nurse home visitation program for high-risk pregnant women that begins at pregnancy and continues until the child’s second birthday. VoorZorg is a version of the NFP that has been translated and culturally adapted for use in Dutch populations. The present study is the first on the effectiveness of this tool to be conducted outside of the United States. Positive effects of VoorZorg on infants’ passive exposure to smoking, breastfeeding and intimate partner violence (IPV), which is a form of child maltreatment, have previously been demonstrated.[12,29] The current study showed that the number of CPS reports was significantly lower among a group of young disadvantaged women who received VoorZorg than in a control group at three years after birth. At 24 months after birth, the intervention group scored higher than the control group on home environment. Furthermore, the prevalence of internalizing behavior was lower among the children of mothers who received VoorZorg. There was no significant difference in the prevalence of externalizing behavior although the trend was in the same direction as for internalizing behavior with less problem behavior in the intervention group. We conclude that the Dutch version of the NFP, VoorZorg, is an effective intervention for young disadvantaged pregnant women that prevents child maltreatment, and improves home environment and child behavioral problems.

### Panel

#### Research in context

##### Systematic review

Medline, PsycINFO, CINAHL, Embase, and the Cochrane library were searched using the (MESH and TiAb) terms "Primary Prevention" OR "Health Education" AND "Child Abuse/prevention and control" AND "Child" for reports published between Jan 1, 2008 and Dec 6, 2013. This strategy identified 171 articles, of which only two were RCTs.[30,31] Furthermore, two systematic reviews using similar search terms for reports published between 1990 and 2007 and between Jan 1, 2000 and July 31, 2008 were included.[32,33] Mikton et al. reported that early childhood home visitation is the most evaluated type of intervention and that there is strong evidence that early home visitation is effective in the prevention of child maltreatment. However, with the exception of Olds’ NFP, for which the effectiveness has been demonstrated, the results in a number of studies are equivocal due to surveillance bias and poor internal validity. The evidence supporting the efficacy of the prevention of child maltreatment for other types of prevention programs is therefore insufficient.[33] Reynolds et al. found that only three programs (i.e., Child Parent Centers, NFP and the Parent Education Program) showed strong evidence for preventive effects in substantiated reports of child maltreatment.[32] Prinz et al. reported that the population-based dissemination of the Triple-P parenting program had positive effects on the prevention of child maltreatment.[30] Zielinsky et al. showed that the NFP appears to prevent child maltreatment early in life and confines first-time reports of neglect to the first four years of life compared to control children from whom CPS reports continued until age 12.[31]

### Interpretation

The aim of the current study was to examine the primary prevention of child maltreatment due to systematic and well-structured home visits by nurses to young disadvantaged pregnant women. In terms of the recommendations and limitations of the studies identified in the above systematic review, our study utilized CPS reports in the evaluation of the intervention as recommended. The validity of these reports may be hampered by surveillance bias and liberal bias. In surveillance, families in the intervention group are visited regularly and therefore the chance to detect child maltreatment is higher than in the control group. And in liberal bias, professionals involved in the intervention tend to report less incidents or only more severe incidents of maltreatment because they are already addressing abuse, neglect, and relevant risk factors in the family or because they do not want to endanger their relationship with the mothers. Other professionals may also be more restrained in reporting to CPS because they are aware that a family is receiving treatment. However, the net effect of surveillance bias and liberal bias differed little in terms of reported incidents, severities and confirmations. [34] The NFP appears to be the first program that is effective in the primary prevention of child maltreatment. The current study adds strong evidence to this conclusion by showing that VoorZorg (i.e., the Dutch equivalent of the NFP) is effective in the prevention of child maltreatment based on official CPS data.

The validity of the use of the prevalence of CPS reports to indicate a reduction in child maltreatment due to home visitations is further supported by the observed improvements in the relevant risk factors for maltreatment, such as the home environment and the children’s behaviors. The reduced prevalence of internalizing behavior at age two appears to be attributable to significant improvements on the somatic complaints and withdrawn subscales. Withdrawn behavior during the elementary school period is associated with severe neglect in early life. It is assumed that neglect leads to insecure attachment relationships that may decrease the children’s capacities to interact successfully with peers. [35] In the Early Start home visitation program, a similar reduction of the prevalence of internalizing behavior at age three was observed, and an additional reduction of parent-reported severe physical assault was also observed. However, no improvement in CPS contacts due to the provision of comprehensive services to high-risk families starting shortly after the birth of the child and lasting for 24 months has been observed for this intervention program. [36] Moreover, somatic complaints are often associated with anxiety disorders in children. [37] It is possible that the reduction in somatic complaints at the age of 24 months is the first indication of a reduction in anxiety and stress in these young children due to the decline in IPV and child maltreatment that resulted from VoorZorg. [12] As high-risk young mothers are often poorly prepared for their role as mothers, the increase in IT-HOME scores at 24 months indicates that the home environments were more enriched and more attuned to the interactional needs of the toddlers due to the home visits by the nurses. The improvements in home environments and the children’s behaviors accord with the reduction in CPS reports because there appeared to be less child maltreatment, more structure and more support for the children in the home-visited families.

A limitation of this study is that we were unable to assess the data from children who were untraceable or had moved to other regions. As each CPS agency only has access to data from the children who have been reported in their region, it is possible that the data from the children who were no longer living in the region were not addressed because this information was not in the database. Furthermore, the CPS agencies only document reports of child maltreatment, which represents only a low percentage of the actual prevalence of incidents of child maltreatment.[38] Nevertheless, the CPS data are considered reliable because these data are based on observations of people other than the parents, which should diminish the bias toward socially desirable answers. A second limitation is that we assumed that each CPS report was a valid case of child maltreatment. In general, 93% of the reports to CPS in the Netherlands appear to be substantiated cases of maltreatment based on subsequent CPS investigations. To examine whether a similar percentage of reports in our study population were substantiated cases of child maltreatment, we requested additional information about the reports from the CPS. We received this additional information for approximately 50% of the children with CPS reports, and 96% of those CPS reports were indeed substantiated cases of child maltreatment (unpublished data). A third limitation is that we had a relatively small number of Turkish and Moroccan participants in our study, which limits the generalizability of this study. Other limitations are that the participants in our study may have been particularly motivated and thus might not be representative of the overall population of disadvantaged mothers. Another limitation is the relatively high dropout in our study, particularly for the IT-HOME and CBCL scales and particularly in the control group. However, the completers and non-completers did not show any difference in patient characteristics which indicates a random missing data situation. The final limitation is that the sample size calculation was conducted in an a priori manner that was based on smoking cessation around the time of childbirth and not on maltreatment. However, we performed a post-hoc calculation on child maltreatment that revealed that the estimated sample-size requirements of these two sample-size calculations were not different.

The results of this RCT of VoorZorg corroborate the positive effects of this type of intervention that have been shown in NFP trials conducted in the US; thus, nurse-home visits represent an efficacious strategy for the primary prevention of child maltreatment. [39] Compared to the costs and the lifetime effects of child maltreatment, VoorZorg is relatively inexpensive. In conclusion, VoorZorg and the NFP are evidence-based programs for the primary prevention of child maltreatment. We recommend that future research examines whether modifications of VoorZorg that tailor the program to the specific needs of families with CPS reports (e.g., the inclusion of the Signs of Safety[40]) can prevent the reoccurrence of child maltreatment.

## Supporting Information

S1 Study Protocol (PDF)

S1 Original Study Protocol (PDF)

S1 CONSORT Checklist (DOC)
