# Supplementary material for: The Effect of VoorZorg, the Dutch Nurse-Family Partnership, on Child Maltreatment and Development: A Randomized Controlled Trial
Source: PLoS One. 2015 Apr 1;10(4):e0120182. doi: 10.1371/journal.pone.0120182 (PMC4382107; doi:10.1371/journal.pone.0120182)
Supplement: S1 Original Study Protocol — (PDF) [file pone.0120182.s002.pdf]

C 1

PROTOCOL WETENSCHAPPELIJK ONDERZOEK:

Ontwikkeling, implementatie en onderzoek naar de effectiviteit van de VoorZorg-interventie middels randomised controlled trial.

Doelstelling

Implementatie en onderzoek naar de effectiviteit van Voorzorg interventie, waarin de risicofactoren worden aangepakt die, tijdens de zwangerschap en in de eerste 2 levensjaren, de lichamelijke, de cognitieve en de gedragsmatige ontwikkeling van nog-te-geboren-worden kinderen van hoog risico moeders bedreigen. Het uiteindelijke doel is om zwangerschaps- en geboorte-uitkomsten te verbeteren voor moeder en kind, om de gezondheid en ontwikkeling van het kind te verbeteren en om de persoonlijke ontwikkeling en mogelijkheden voor opleiding en werk voor de moeder te verbeteren wat zal leiden tot een betere moeder-kind interactie.

Inleiding

Preventieve interventies tijdens de zwangerschap die risico-factoren elimineren of reduceren die de lichamelijke, cognitieve en gedragsmatige ontwikkeling van kinderen bedreigen, kunnen een beslissend en langdurig positief effect hebben op de ontwikkeling van kinderen, met name wat betreft reductie van probleemgedrag. Bekende risico-factoren zijn o.a. roken, alcohol gebruik, drugs gebruik, slecht dieet, slechte lichamelijke gezondheid, geestelijke problemen bij moeders, misbruik of verwaarlozing in het verleden, tiener zwangerschap en lage opleiding. Van de ouders die onder matige sociaal-economische omstandigheden leven bestaan bij 3% meerdere risico-factoren tegelijkertijd en hun kinderen hebben risico op een achterstand in hun lichamelijke, cognitieve, gedragsmatige en emotionele ontwikkeling. Om echt een verschil te maken in de levens van kinderen die onder dergelijke omstandigheden moeten opgroeien, zouden interventies zich moeten richten op de bekende risico-factoren gedurende sensitieve periodes voor de ontwikkeling, zoals zwangerschap en vroege kindertijd.

In twee randomised controlled trials, de 'Elmira-studie' en de 'Memphis-studie' vond Olds (1998) significant positieve gezondheidseffecten tijdens zwangerschap en eerste levensjaar alsook gedurende kindertijd en adolescentie in deze populatie van hoog-risico moeders en kinderen met lage sociaal-economische situatie. De vermindering van sigaretten roken en de verbetering van het dieet resulteerden in een verbeterde intra-uteriene ontwikkeling en een verbeterde cognitie, hanteerbäarder temperament en een reductie van agressief probleemgedrag later. De invloed op kindermishandeling was het gevolg van de aandacht in het programma voor een sensitieve en responsieve interactie met het kind. Hierdoor trad ook een verbetering op van het temperament van het kind waardoor het makkelijker hanteerbaar en daardoor meer belonend was voor deze hoog-risico moeders om met hun kinderen om te gaan.

De Nurse Family Partnership interventie, ontwikkeld door David Olds en in Nederland genoemd VoorZorg interventie, is inmiddels vertaald, aangepast en op kleine schaal toegepast bij enkele moeders; handleiding en trainingsmaterialen zijn beschikbaar en de ervaring en expertise om de interventie in de Nederlandse Jeugdgezondheidszorg toe te passen is groeiende. Moeders zijn geïncludeerd en hebben het VoorZorg-programma ondergaan; de eerste baby's zijn geboren en zijn gestart met borstvoeding. De eerste fase van het project is afgerond en de voorbereidingen voor de tweede fase moeten worden genomen.

In de Jeugd(gezondheids)zorg zijn vele interventies ontwikkeld, een aantal op effectiviteit onderzocht en slechts enkelen daadwerkelijk effectief gebleken (Veelbelovend en

Effectief, Rapport van de Inventgroep). Het is verstandig is om interventies in te zetten waarvan het effect is vastgesteld.

Van begin af aan heeft de projectgroep zich gecommitteerd om aansluitend aan de ontwikkeling van de VoorZorg interventie ook toe te werken naar de uitvoering van een trial om de effectiviteit vast te stellen. Uiteindelijk is het de bedoeling dat de interventie die aangeboden wordt aan deze hoog-risico moeders daadwerkelijk een positief effect heeft op de ontwikkeling van hun kinderen; de risicofactoren die de ontwikkeling negatief beïnvloeden moeten worden gereduceerd en de factoren die een gezonde ontwikkeling bevorderen moeten worden onderstcund. Onderzocht wordt via welke mechanismen effecten bereikt worden om toekomstige interventies daar op af te kunnen stemmen alsook na te gaan welke organisatorische voorwaarden nodig zijn om de interventie gedegen uit te voeren.

Ontwikkeltraject

In het Voorzorg programma worden hoog-risico zwangere vrouwen regelmatig bezocht door verpleegkundigen tijdens de zwangerschap, rondom de geboorte, na 6 maanden en 1 en 2 jaar na de geboorte. Tijdens de bezoeken, bekijken de verpleegkundigen (1) veranderingen in het gedrag van de moeders om de gezondheid en ontwikkeling van het kind te stimuleren en om de leef-omstandigheden van moeders te verbeteren met de nadruk op dieet en lichamelijke gezondheid en de reductie van drugsgebruik tijdens de zwangerschap, (2) de kwaliteit van steunende relaties, en (3) gebruikmaking van regulaire hulpverlening door de moeders.

Het ontwikkeltraject van dit onderzoek is inmiddels bijna afgerond. Hierin werd gestart met de aanpassing van de originele Home Visitation Interventie, zoals ontwikkeld door dr. David Olds, voor gebruik in Nederland. Condities zijn geformuleerd waaronder het programma wordt gebruikt in Nederland in overleg met dr. David Olds. Twee Nederlandse programma-ontwikkelaars zijn getraind aan het Denver Prevention Research Center. De originele interventie is vertaald en aangepast rekening houdend met de specifieke Nederlandse sociale en culturele context. Ten behoeve van deze aanpassing is een analyse gedaan van beïnvloedbare factoren op gedrags- en leefstijlnivo (Bakker's Balans-model, PREFFI, HBM). De aanwezige kennis en ervaring betreffende de implementatie van interventies bij deze notoir moeilijke doelgroep van jonge moeders is gebundeld in een werkgroep.

Een screeningsprocedure is ontwikkeld om hoog-risico moeders vroeg in de zwangerschap te kunnen identificeren. Vanuit jeugdhulpverlening en kinderpsychiatrie is bekend dat ongeveer 10% van de moeders tot de hoog risicogroep behoren, dat wil zeggen dat zij problemen ervaren in het opvoeden en zorgdragen van hun kind. De werkgroep heeft een praktische en goed hanteerbare in- en exclusielijst ontwikkeld voor gebruik aan het begin van de zwangerschap.

Een kleinschalig, lokaal implementatieproject is uitgevoerd bij 2 zorgorganisaties (Evean Jeugdgezondheidszorg te Zaanstraak/Waterland en Centrum Ouder en Kindzorg te Rotterdam). Het Trimbos instituut heeft de huisbezoeken geëvalueerd en een aantal conclusies en aanbevelingen gedaan. Deze zijn samengevat: de beoogde doelgroep is bereikt, moeders hadden veel steun aan de Voorzorg-verpleegkundige en de ondersteunende materialen voldeden grotendeels aan de behoeften van de moeders en de verpleegkundigen.

Relevantie

Gebrekkige of gevaarlijke opvoed-situaties, niet in staat zijn om kinderen op te voeden en kindermishandeling komen regelmatig voor in Nederland, te vaak als we kijken naar recente incidenten. Betrouwbare data over de incidentie van mishandeling en verwaarlozing ontbreken, en de beschikbare data zijn slechts het topje van de ijsberg. Geschat wordt dat in Nederland jaarlijks 50.000 tot 80.000 kinderen lichamelijk of emotioneel verwaarloosd

worden. Ongeveer 50 van deze kinderen sterven jaarlijks als gevolg van mishandeling of verwaarlozing door de ouders. Elk jaar bellen meer mensen naar de 'Advies en Meldpunten Kindermishandeling'. In 2003 werden 28.000 meldingen geregistreerd; de werkelijke incidentie is waarschijnlijk 2 keer zo hoog.

De consequenties van verwaarlozing en kindermishandeling zijn vaak het hele verdere leven merkbaar en deze kinderen lopen het risico om later zelf ook slecht presterende ouders te zijn die hun kinderen verwaarlozen of mishandelen. Kindermishandeling is een belangrijke risico-factor voor lage schoolprestaties, spijbelen en voortijdig schoolverlaten, voor oppositioneel en agressief gedrag en voor crimineel gedrag. Het is ook een risico-factor voor ernstige lichamelijke klachten. De kosten van kindermishandeling voor de maatschappij zijn hoog.

Laag geschoolde vrouwen met een laag inkomen en vele additionele risico's hebben een grote kans om kinderen te baren wiens intra-uteriene ontwikkeling al gecompromitteerd was met mogelijke schade bij de geboorte. Als gevolg daarvan zijn ze moeilijker op te voeden, wat opgeteld bij de opvoedkundige onkunde van de ouders, het risico voor verwaarlozing en mishandeling groter maakt. Actieve, intensieve en goed gestructureerde ondersteuning van deze hoog-risico moeders tijdens de zwangerschap zal waarschijnlijk een aantal risico-factoren doen afnemen (slecht dieet, alcohol en roken, drugsgebruik en stress), waardoor het risico op slechte geboorte uitkomsten reduceert (laag geboorte gewicht, pre- en dysmaturiteit, hersenschade), wat het risico op opvoedkundige onkunde bij ouders reduceert (slaan en schoppen, verwaarlozing, slechte voeding), waardoor een aantal factoren worden gestimuleerd zoals de ouder-kind binding, vertrouwen in opvoeding en gebruikmaking van professionele en sociale hulp.

Randomised Controlled Trial

Vraagstellingen

1. Welk effect heeft de Voorzorg interventie op risicofactoren tijdens zwangerschap en op – korte termijn - uitkomsten rondom geboorte en de eerste 2 levensmaanden?
2. Welk effect heeft de Voorzorg interventie op de ontwikkeling van kinderen en hun moeders gedurende de eerste twee levensjaren?
3. Welke voorwaarden zijn noodzakelijk voor een optimale implementatie en hoe kosten-effectief is de interventie?

Ad 1: Welk effect heeft de Voorzorg interventie op risicofactoren tijdens zwangerschap en op – korte termijn - uitkomsten rondom geboorte en de eerste 2 levensmaanden?

Nagegaan wordt of en in welke mate tijdens de zwangerschap verbetering is opgetreden op een van de 6 gebieden waar de Voorzorg interventie zich op richt (persoonlijke gezondheid, gezonde omgeving, levensloop, moederschap, familie en vrienden, en gebruik van voorzieningen). Nadruk wordt gegeven aan (zwangerschap gerelateerde) gezondheidsklachten en zwangerschapscomplicaties, dieet, gebruik van alcohol, sigaretten en andere middelen, alsook aan de geestelijke gezondheid – m.n. angsten en depressieve klachten – van de moeder. Gekeken wordt naar omgevingsfactoren zoals financiën, huisvesting, mishandeling en verwaarlozing. Ook wordt gevraagd naar schoolloopbaan of werk, naar de voorbereidingen voor de bevalling en de komst van het kind, naar het netwerk alsook of mensen bereid zijn de aanstaande moeder te begeleiden bij de bevalling en kraamperiode. Tenslotte wordt het gebruik van gezondheidsvoorzieningen nagevraagd.

Nagegaan wordt of en in welke mate rondom geboorte en kraamperiode een verbetering is opgetreden. Daartoe wordt de verslaglegging over geboorte en kraamperiode nagetrokken op bijvoorbeeld vroeggeboorte, complicaties, borstvoeding of flessenvoeding alsook eventuele problemen bij het op gang brengen van de borstvoeding. Vanzelfsprekend worden geboortegewicht, Apgar-score, en Hb vergeleken. Ook wordt nagegaan of sprake is geweest van 'kraamtranen' en depressieve klachten van de moeder op 2 maanden.

T.a.v. deze uitkomsten worden in eerste instantie hoofdeffecten geanalyseerd. Olds heeft alle effecten nader bekeken voor tienermoeders vs oudere moeders en voor rokers vs niet-rokers – effecten werden m.n. gevonden voor de tienermoeders en voor de moeders die rookgewoonten wisten te reduceren tijdens de zwangerschap. Voor de Voorzorg trial wordt toegevoegd dat alle effecten ook voor mishandelde/verwaarloosde vs niet verwaarloosde en voor agressieve vs niet agressieve moeders geanalyseerd kunnen worden – dit kan effecten aantonen van Voorzorg op angsten tijdens de zwangerschap, affectieve problemen na de geboorte, stress, beschikbaarheid van netwerk.

Ad 2: Welk effect heeft de Voorzorg interventie op de ontwikkeling van kinderen en hun moeders gedurende de eerste twee levensjaren?

Nagegaan wordt of en in welke mate een positief effect aanwezig is op het temperament en regulatiestoornissen, vroege gedragsproblemen, ongelukken en incidenten, SEH-bezoeken bij het kind, alsook op de lichamelijke groei en ontwikkeling en op de kwaliteit van de hechting. De kwaliteit van de opvoeding m.n. inzake veiligheid, sensitiviteit en responsiviteit, de lichamelijke en geestelijke gezondheid van de moeder, en de perceptie van de moeder van haar kind m.n. betreffende temperament. Voorts wordt gelet op de kwaliteit van de voeding, middelengebruik van de moeder, haar sociale netwerk, plannen tav scholing en werk, financien en huisvesting, gezinsplanning en gebruik van anticonceptie. Ook wordt aangesloten bij de (groei in) vaardigheden van de moeders op de 6 programmadomeinen.

T.a.v. deze uitkomsten worden in eerste instantie hoofdeffecten geanalyseerd. Olds heeft gevonden dat een belangrijk effect van de interventie gemedieerd wordt door de afname van het aantal sigaretten dat moeders roken. Ook vond hij dat moeders' sense of control een moderator was voor effecten op mishandeling en SEH-bezoek – moeders die het gevoel hebben weinig controle te hebben mishandelen vaker en moeten vaker de SEH bezoeken, terwijl de interventie voor alle moeders een reductie van mishandeling en SEH-bezoek laat zien. Belangrijk voor nu en zeker voor toekomstige analyses is om meerdere metingen van de neuromotore ontwikkeling en prikkelbaarheid/temperament te verrichten.

Ad 3: Welke voorwaarden zijn noodzakelijk voor een optimale implementatie en hoe kosten-effectief is de interventie?

Om moeders op een verstandige manier met zwangerschap en baby te laten omgaan (reductie van risicofactoren!) moeten verpleegkundigen deze moeders optimaal informeren, instrueren en motiveren; om dit te kunnen moeten de verpleegkundigen tevoren goed getraind worden alsook tijdens de interventie goed begeleid worden door hun supervisor; om dit te kunnen moeten trainers en supervisoren over voldoende en gedegen trainingsmateriaal en expertise beschikken. Dit getrapte implementatiemodel toont de complexiteit van de interventie en maakt inzichtelijk dat op diverse niveaus fors ingezet moet worden om de kwaliteit van implementatie te verzekeren. Rondom deze vraagstelling worden twee aspecten

onderscheiden: (1) hoe adequaat kunnen verpleegkundigen de interventie zoals bedoeld uitvoeren en (2) welke kosten moeten daartoe gemaakt worden. In de Jeugd(gezondheids)zorg zal, net als in andere gebieden van de geneeskunde, in toenemende mate gevraagd worden naar de kosten die gemaakt moeten worden om gezondheidseffecten te bereiken vandaar dat deze studie van een Kosten Effectiviteits Analyse (KEA) voorzien wordt waarbij de kosten van het gebruik van zorgvoorzieningen worden meegenomen. De trial sluit in dit opzicht aan bij de doelstellingen van de 'impuls opvoed- en gezinsondersteuning.'

Implementatie wordt o.a. gevolgd met het registratiesysteem waarbij per huisbezoek geregistreerd wordt hoeveel procent van de tijd aandacht besteed is aan de doelen van dat bezoek. Economische kosten worden verzameld op het niveau van de verpleegkundige en de instelling. Feitelijke kosten worden geschat vanuit een samenlevingsperspectief en omvatten de organisatie en training, de feitelijke tijd besteed aan interventie, voorbereiding en vervoer, registratie, begeleidende activiteiten zoals overleg met andere professionals, supervisie, etc. De belangrijkste data die verzameld moeten worden betreffen de uren gemaakt door verpleegkundigen, supervisoren, trainers en anderen, en de kosten van handleidingen en trainingen. Ook wordt gekeken naar de kosten van het gebruik van zorgvoorzieningen in controle- en interventieconditie. Verschillende kostenscenario's zullen worden onderzocht waarbij enerzijds rekening kan worden gehouden met de problematiek die wordt aangetroffen en anderzijds met verschillen in organisaties. Naast de kostenregistratie moet vanzelfsprekend ook een registratie van de lichamelijke en geestelijke gezondheid en van zorggebruik in controle- en interventieconditie plaatsvinden. Uitgezocht wordt of het mogelijk is om uitkeringen e.d. te registreren.

Design

Het design van de studie is gebaseerd op een trial met minimaal 456 geselecteerde zwangere hoog-risico moeders die at random aan controle of interventie-conditie worden toegewezen. Van belang daarbij zijn de selectie en inclusie, de randomisatie-procedure, de power van de studie, de controle en interventieconditie, de organisatie van de metingen in tijd en naar inhoud, de analyses.

Selectie en inclusie

Verloskundigen en andere professionals (huisartsen, gynecologen, street corner werkers, ea) zullen zwangere vrouwen screenen op de volgende criteria: geen eerder geboren kind (een aantal zwangeren onderging eerder een abortus), zwangerschapsduur maximaal 28 weken, maximaal VMBO-P afgerond, enige beheersing van de Nederlandse taal.

Deze moeders wordt gevraagd of zij instemmen dat zij door de VoorZorg-verpleegkundige bezocht worden die met hen in gesprek zal gaan over een aantal aanvullende risicofactoren. Vervolgens screent de VoorZorg verpleegkundige de a.s. moeders op aanwezigheid van een of meerdere van de volgende criteria: geen (steunend) sociaal netwerk / partner, alcohol- en/of drugsgebruik, actueel gezinsgeweld of partnergeweld, zelf mishandeld in haar jeugd, psychische problematiek: angst, depressie, irreële opvattingen over a.s. moederschap, 'niet-pluis' gevoel bij verpleegkundige, niet afgemaakte vooropleiding, schoolverlater, werkloos, financiële en/of huisvestingsproblemen.

Tenslotte moeten a.s. moeders aan de volgende voorwaarden voldoen: ze moeten de insteek van het programma lijken te begrijpen, ze moeten geen verhuisplannen buiten de regio hebben voor de komende 2 jaar, ze moeten bereid zijn om aan het hele programma mee te doen, ze moeten accoord gaan met random toewijzing aan controle of interventie conditie, en ze moeten willen deelnemen aan het onderzoek. Vrouwen die in een asielzoekersprocedure zitten worden uitgesloten van de trial daar het inherent is aan deze procedure dat ze per direct

moeten verhuizen; vrouwen met een vluchtelingenstatus komen wel voor inclusie in aanmerking.

Zwangeren worden gerecruteerd in verschillende gebieden in Nederland. De trial wordt uitgelegd aan deze aanstaande moeders en informed consent verkregen. Uiteindelijk besluit de VoorZorg-verpleegkundige in overleg met haar supervisor of de moeder in aanmerking komt voor deelname aan de interventie; de redenen voor inclusie worden geregistreerd. De gegevens van de aanstaande moeder worden doorgegeven aan het onderzoeksteam op het VUmc. Moeders kunnen zich te allen tijde aan het onderzoek naar de VoorZorg-interventie onttrekken.

Randomisatie

Zodra een moeder in aanmerking komt voor deelname aan de studie zal de studiecoördinator in de vooraf gemaakte digitale randomisatietabel nagaan of de moeder aan de interventie- of controleconditie wordt toegekend. Randomisatie zal gebeuren op het niveau van de moeders binnen een regio om te garanderen dat alle regio's in dezelfde mate deelnemen en te voorkomen dat de regio's als confounder zullen werken (sociaal-economische en etnische samenstelling van de regio, kwaliteit van de jeugd(gezondheids)zorg in een regio); gestratificeerd wordt ook op etniciteit (geen of wel 1^e^ of 2^e^ generatie migrant – Turks, Marokkaans, Surinaams/Antilliaans, Kaap-Verdiaans, overigen). Achtergrond is de grote invloed van etniciteit op risicofactoren en opvoedstijlen; migranten kennen een ander risicoprofiel (weinig roken en alcohol, erg laag opleidingsniveau) hetgeen consequenties heeft voor uitvoering en resultaten van de trial. Vrouwen die in hetzelfde huishouden wonen als vrouwen die reeds geïncludeerd zijn worden aan dezelfde conditie toegewezen teneinde diffusie van conditie/interventie te voorkomen.
Randomisatie geschiedt door de projectleiding teneinde hieraan strikt vast te houden.

Interventie en controle conditie

De zwangeren die in aanmerking komen voor de controle conditie worden naar de verloskundige of de verwijzer terugverwezen en zullen de gebruikelijke zorg ontvangen. Met gebruikelijke zorg bedoelen we de zorg die op dat moment lokaal voor deze groep moeders geboden wordt. Het is nadrukkelijk niet de bedoeling dat verpleegkundigen die moeders uit de controle-conditie begeleiden 'dunnetjes' VoorZorg gaan doen. Ook zullen de moeders uit de controle-conditie niet in het VoorZorg-dossier worden opgenomen. Vrouwen in beide condities ontvangen een vergoeding voor de tijd die het interview hen kost als erkenning en vergoeding voor deelname aan de trial.

De vrouwen die deelnemen aan de VoorZorg interventie ontvangen deze tijdens de zwangerschap en het eerste en tweede jaar. De interventie zal verzorgd worden door verpleegkundigen van de jeugdgezondheidszorg organisaties die getraind en gelicenseerd zijn om de interventie te implementeren en die toegezegd hebben voldoende ondersteuning en supervisie te bieden om adequate implementatie te verzekeren.

Power

Ten einde voldoende power te hebben zullen minimaal 228 zwangere vrouwen en een corresponderend aantal vrouwen in de controle-conditie minstens de zwangerschapscomponent van de VoorZorg-interventie doorlopen moeten hebben. Met deze aantallen zwangeren kunnen hoofdeffecten op de belangrijkste uitkomst variabelen rond geboorte, eerste en tweede levensjaar worden vastgesteld. De Amerikaanse onderzoekers hanteerden een zelfde aantal zwangeren en waren uiteindelijk in staat om effecten tot minimaal het 15e levensjaar vast te stellen. Of met deze aantallen ook interactie-effecten kunnen worden vastgesteld moet bezien worden. Wanneer daar mogelijkheden voor zijn

kunnen meer moeders geïncludeerd worden, maar dit is afhankelijk van de financiering van de interventie en van de financiering van de metingen.

Twee benaderingen zijn gevolgd t.b.v. de power-berekening. Allereerst werd geprobeerd een power-berekening uit te voeren, maar daarvoor moeten gegevens over prevalentie, spreiding en te verwachten effecten bekend zijn terwijl deze gegevens voor onze populatie van hoog-risico moeders niet echt beschikbaar zijn. Twee voorbeelden werden uitgewerkt: reductie van sigarettenroken tijdens zwangerschap- een belangrijke intermediaire uitkomst, afname van meldingen van mishandeling en verwaarlozing op 15-jarige leeftijd – een belangrijke langc-termijn doelstelling van het programma. Uitgaande van 30% rokende vrouwen die gemiddeld 4 sigaretten minder roken a.g.v. interventie (in VS werd een gemiddelde reductie van 5 sigaretten vastgesteld) kunnen bij een standaard deviatie van 12 nog kleine tot middelmatige effecten vastgesteld worden met een power >.80. Uitgaande van een gemiddelde reductie van het aantal gerapporteerde gevallen van kindermishandeling en verwaarlozing op 15-jarige leeftijd is de power om een klein effect te vinden .99 bij eenzijdige toetsing.

Als tweede hebben we gekeken naar de prevalentie van risicofactoren in de Nederlandse bevolking. Belangrijke factoren die aangepakt worden door de interventie opereren in Nederland minstens op hetzelfde of zelfs op een hoger niveau in vergelijking tot de Amerikaanse gegevens. Tweemaal zoveel vrouwen roken terwijl het aandeel van de zware rooksters zelfs hoger is in Nederland; overall 25% en tijdens de zwangerschap 21%, van de 16-jarige meisjes rookt 36%; alcohol-consumptie is tweemaal zo hoog, aandachtsproblemen en hyperactiviteit – aanwijzing voor een moeilijk temperament – liggen op hetzelfde tot een iets hoger niveau in vergelijking tot de VS; niveau's van sexueel misbruik, mishandeling en verwaarlozing worden verondersteld op eenzelfde niveau te liggen (Baartman, persoonlijke mededeling). Op bevolkingsniveau zijn in Nederland minder tienerzwangerschappen, maar met de gehanteerde inclusiecriteria zal een aanzienlijk deel van de vrouwen die VZ ontvangen uit tieners bestaan.

Organisatie van het experiment in de tijd

Zwangere vrouwen zullen geïncludeerd worden tussen de 16 en 28 weken zwangerschapsduur; geboorte, eerste en tweede verjaardag van het kind zijn vastgestelde momenten. De interventie wordt uitgevoerd over de gehele onderzoeksperiode (zwangerschap, eerste en tweede jaar). Metingen vinden plaats bij opname in de studie, op 32 weken zwangerschap, rond de geboorte, rond zes maanden, rond eerste en tweede verjaardag. Metingen vinden plaats door middel van een interview en door registratie vanuit de bestaande dossiers. Vragenlijsten worden zo min mogelijk gebruikt vanwege de te verwachten taal- en leesproblemen. Als interviewers zullen vrouwen worden geselecteerd met een medische, verpleegkundige of pedagogische achtergrond die in staat zijn de aansluiting te maken met de vrouwen die aan de trial deelnemen. Aangezien alle vrouwen geacht worden voldoende Nederlands te beheersen om aan de interventie deel te kunnen nemen zullen niet speciaal allochtone interviewsters geworven worden. De interviewsters worden intensief getraind en zullen niet geïnformeerd worden over de interventieconditie (wel/geen iv). Informatieverzameling voor het onderzoek wordt door daarvoor getrainde interviewsters gedaan; het is niet de bedoeling dat de verpleegkundigen het onderzoek gaan uitvoeren.

Bij aanvang worden vrouwen geïnterviewd over hun lichamelijke conditie (ziektes, dieet, sigaretten en alcoholconsumptie, etc.) emotionele (gevoelens van angst en depressie, een geschiedenis van mishandeling en verwaarlozing), relationele (huidige partner, sociale steun) en sociale determinanten (onderwijs, financiële problemen, huisvesting, gebruik van bestaande gezondheidszorg). Moeders' sense of control over de omstandigheden wordt

vastgesteld. Partners wordt gevraagd om over emotionele en gedragsproblemen te rapporteren (YASR).

Gedurende de gehele studie worden metingen over het gewicht, borst of flesvoeding, groei volgens het van Wiechen schema, zoals verzameld door de reguliere zorgverleners (vroedvrouwen, consultatiebureau) opgenomen in de studie. Ook worden gegevens van bevalling of kraamperiode overgenomen uit het dossier van de kraamzorg en verloskundige. Het is de bedoeling de gegevens te verzamelen via een elektronisch cliënten dossier ontwikkeld voor gebruik in de Nederlandse jeugdgezondheidszorg (bijvoorbeeld MLCAS), maar wellicht behoort dit thans nog niet tot de mogelijkheden.

Tijdens de zwangerschap wordt minstens een meting van lichamelijk (roken, alcohol, middelen gebruik), emotioneel (angst en depressie, sociale steun), sociaal (gebruik van gezondheidszorg voorzieningen) functioneren verzameld. Voorts wordt eenmaal een urine sample verzameld om urineweginfecties na te gaan; urineweginfecties kunnen namelijk een vroeg-geboorte uitlokken.

Omstreeks en tijdens de geboorte worden lichamelijke (vroeggeboorte, hypertensie, APGAR score, navelstreng bloed) en emotionele (kraamtranen, sociale steun, intentie om borstvoeding te geven) metingen verzameld. Voorts word aan de partners gevraagd om hun betrokkenheid bij bevalling en opvang van het kind aan te geven.

Op zes maanden wordt de ontwikkeling van het kind gemeten, ook wordt gelet op tekenen van prikkelbaarheid (problemen rondom slapen, voeding, huilen) en of de moeder haar kind kan troosten; dit als vroege uitingen van het temperament van het kind.

Aan het einde van het eerste jaar wordt opnieuw de ontwikkeling van het kind vastgelegd. De moeder-kind interactie wordt gemeten tijdens het verschonen van de luiers en tijdens een voedingsmoment. Moeders worden geïnterviewd over hun lichamelijke, emotionele, relationele en sociale functioneren alsook over agressieve momenten in het gezin. De thuissituatie wordt geobserveerd ten aanzien van de veiligheid voor het kind, de beschikbaarheid van voedsel en fruit, en de beschikbaarheid van speel materialen.

Aan het einde van het tweede jaar wordt opnieuw de ontwikkeling van het kind vastgesteld. Onafhankelijke rapportages over misbruik en verwaarlozing (thuis observaties, zorgregistraties) worden verzameld. Moeders worden geïnterviewd over dieet, roken en alcoholgebruik, ongelukken, bezoeken aan spoedeisende hulp en hospitalisatie. Voorts worden de financiën, de huisvesting en onderwijs vastgesteld, alsook het gebruik van anticonceptie en eventuele zwangerschappen. Gelet wordt op de stabiliteit van de relatie en de betrokkenheid van de vader; ook wordt gevraagd naar psychopathologie bij de moeder waaronder depressieve klachten en agressiviteit. De thuissituatie wordt geobserveerd ten aanzien van de veiligheid, de beschikbaarheid van voedsel en fruit, en de beschikbaarheid van speel materialen. De metingen worden gecoördineerd met de metingen van het OKE-project en met andere projecten over de vroege vaststelling van een problematische ontwikkeling (TNO ULMC, Leiden); mn wordt geprobeerd de suggesties van de Invent-groep over te nemen en aan te sluiten bij lopend onderzoek in Duitsland.

Tijdens de interventie rapporteren de verpleegkundigen het aantal sessies, het aantal minuten dat besteed wordt aan verschillende onderdelen van het programma, het aantal no-shows en de moeders die het programma verlaten. De supervisie geeft per sessie aan in welke mate de verpleegkundige de beoogde onderwerpen heeft behandeld.

Gebieden VZ interventie	Inclusie 16 – 28 weken		Zwangerschap (meting onderzoek 32 weken)	
	Inventarisatie risicofactoren	Instrumenten	variabelen	Instrumenten
Persoonlijke gezondheid	- verloop zwangerschap	- vragenlijst 'gezondheid van de moeder' (RF, 1^e huisbezoek, deel A)	- verloop zwangerschap	- gegevens gewichtstoename via reguliere zorg
	- geestelijke gezondheid	- zelfrapportage emot. & gedragsproblemen (YASR), rapportage moeder door oma (YABCL, verkorte versie), rapportage partner door moeder (YABCL, verkorte versie).	- geestelijke gezondheid	- depressie: EPNDS
	- lichamelijke gezondheid	- SF 36	- middelengebruik	- urine monster
	- middelengebruik	- vragenlijst Middelgebruik Moeders (GR-studie)		
	- 'sense of control'/ self-efficacy	- Utrechtse Coping Lijst (UCL-19)<br>- vragenlijst 'gezondheid van de moeder' (RF, 1^e huisbezoek, deel B)	- 'sense of control'/ self-efficacy	- vragenlijst 'gezondheidsgewoonten' (RF, 36 wk.)
	- prenatale stress *	- STAI-trait<br>- angst in de zwangerschap (Huizink)	- prenatale stress *	- daily Hasles (APL)<br>- life events
Gezonde omgeving	- financiën<br>- huisvesting<br>- wijk	- vragenlijst 'demografische gegevens' (RF)	- financiën<br>- huisvesting	
Levensloop	- schoolloopbaan<br>- werkloosheid	- vragenlijst 'demografische gegevens' (RF)	- schoolloopbaan<br>- werkloosheid	
	- mishandeling/verwaar-lozing in verleden			
	- actueel gezinsgeweld of partnergeweld	- vragenlijst 'relaties met familie en vrienden' (RF, 36 wk.)<br>- 'Revised Conflict Tactics Scales (CTS2)	- actueel gezinsgeweld of partnergeweld	- vragenlijst 'relaties met familie en vrienden' (RF, 36 wk.)
Moederschap	- opvattingen over het moederschap	- 3-min. speech sample	- opvattingen over het moederschap	- 3-min. speech sample
	- voorbereiding voor bevalling en komst van kind	- Maternal (ev. ook Paternal) Antenatal Attachment Scale	- voorbereiding voor bevalling en komst van kind	
Familie en vrienden	- bestaande netwerk<br>- ervaren steun<br>- afwezigheid partner		- bestaande netwerk<br>- ervaren steun<br>- afwezigheid partner	- vragenlijst 'demografische gegevens' (RF, $1^e/2^e$ huisbezoek)
Gebruik van voorzieningen	- inventarisatie gebruik	- tabel RF formulier	- inventarisatie gebruik	- verwijsformulier RF (continu bijgehouden)

RF = Registratieformulieren VoorZorg

** Prenatale stress:*
- Pregnancy Related Anxieties Questionnaire-Revised/ Vragenlijst over bezorgdheid in de zwangerschap (Van den Bergh, 1989; Huizink, 2000)
- Perceived Stress Scale/ Vragenlijst voor ervaren stress (Cohen & Williamson, 1987)
- Alledaagse problemenlijst (Vingerhoets)

Gebieden VZ interventie	Rondom geboorte en kraamperiode		Gedurende hele postnatale periode	
	variabelen	Instrumenten	variabelen	instrumenten
Persoonlijke gezondheid	- Gezondheid kind<br><br>- Psychische gezondheid moeder	- 'baby-geboorteformulier' RF<br>- natrekken reguliere verslaglegging geboorte en kraamperiode*<br>- verzameling navelstrengbloed<br>- neurologisch onderzoek	- gezondheid kind	- via reguliere zorgverlening elektronisch dossier: MLCAS, o.a. groei: van Wiechen schema
Gezonde omgeving				
Levensloop				
Moederschap				
Familie en vrienden	- Betrokkenheid partners			
Gebruik van voorzieningen				

* geboortegewicht, Apgar-score, Hb, vroeggeboorte, complicaties, borstvoeding/flessenvoeding, problemen op gang komen borstvoeding.

Vragenlijst Demografische gegevens (RF): bij leeftijd kind ± 6, 12, 18 en 24 maanden
Vragenlijst 'gezondheidsgewoonten' RF: kind 1 jaar
Vragenlijst 'relaties met familie en vrienden' RF: kind 1 jaar
Baby gezondheidsformulier RF: 6 maanden

Meetmomenten: 2 maanden, 6 maanden, 12 maanden, 24 maanden

<u>Meetmoment: 2 maanden</u>

kind		moeder	
Temperament		Lichamelijke gezondheid moeder	
Regulatiestoornissen	GR Vragenlijst 'The baby Questionnaire'	Geestelijke gezondheid moeder	- Kraamtranen: 11 vragen<br>- Postnatale depressie: EPNDS
Vroege gedragsproblemen		Kwaliteit opvoeding	
Ongelukken/incidenten/ SEH bezoeken		Moederschap	
Thuissituatie		Levensloop	
Ontwikkeling			Video borstvoeding

Meetmoment: 6 maanden

kind		moeder	
Temperament	IBQ (Rothbart)	Lichamelijke gezondheid moeder	
Regulatiestoornissen		Geestelijke gezondheid moeder	
Vroege gedragsproblemen		Kwaliteit opvoeding	
Ongelukken/incidenten/ SEH bezoeken		Moederschap	
Thuissituatie	HOME	Levensloop	
Ontwikkeling	Bailey Scales Infant Development (BSID II) (motorische en mentale ontwikkeling)		

Meetmoment: 12 maanden

Kind		moeder	
Temperament	IBQ (Rothbart)	Lichamelijke gezondheid moeder	
Regulatiestoornissen		Geestelijke gezondheid moeder	
Vroege gedragsproblemen		Kwaliteit opvoeding	
Ongelukken/incidenten/ SEH bezoeken		Moederschap	Video verluieren
Thuissituatie	HOME	Levensloop	
Ontwikkeling	Activiteiten die u met uw kind onderneemt		

Meetmoment: 24 maanden

Kind		moeder	
Temperament	IBQ (Rothbart)	Lichamelijke gezondheid moeder	
Regulatiestoornissen		Geestelijke gezondheid moeder	Nijmeegse ouderlijke stress index (NOSI, '92)
Vroege gedragsproblemen	Gedragsvragemlijst CBCL 1,5 jr<br>Taal op lft 1 jr.	Kwaliteit opvoeding	Harsh parenting<br>Opvoeden
		Moederschap	
Ongelukken/incidenten/ SEH bezoeken		Levensloop	
Thuissituatie	HOME	Gebruik van voorzieningen	- tabel RF formulier
Ontwikkeling			

Analyses
Analyses worden uitgevoerd het behulp van chi-square statistiek en variantieanalyses waarbij moeders en kinderen die de interventie ontvangen vergeleken worden met de moeders en kinderen in de controle conditie. In vervolg analyses worden confounders of determinanten met behulp van multivariate statistieken bekeken. Omdat de periode waarover moeders en kinderen gevolgd worden gering is en een aantal van de concepten van aard veranderd zal het nu niet mogelijk zijn groeimodellen te gebruiken.

Migranten populaties
Thans kan het VoorZorg programma aangeboden worden aan moeders die in enige mate Nederlands kunnen spreken. De meeste moeders die in Nederland verblijven kunnen daarom in het programma worden geïncludeerd.

Verzekering
Ingevolge art. 7 van de Wet medisch -wetenschappelijk onderzoek met mensen (Staatsblad 1998, 161) is door de verrichter van het onderzoek, het VUmc, een verzekering afgesloten die de door het onderzoek veroorzaakte schade door dood of letsel van de deelnemende proefpersonen dekt. Deze verzekering is afgesloten bij Onderlinge Waarborgmaatschappij Centramed b.a. , Postbus 191, 2270 AD Voorburg. De verzekeraar en de verzekering voldoen aan het besluit verplichte verzekering bij medisch-wetenschappelijk met mensen (Staatsblad 2003, 266) gestelde eisen. Aan het onderzoek deelnemende proefpersonen zullen schriftelijk worden geïnformeerd over de verzekering.

Referenties

- Olds DL, Henderson CR, Tatelbaum R, Chamberlin R. Improving the delivery of prenatal care and outcomes of pregnancy: A randomized trial of nurse home visitation. Pediatrics, 1986;77:16-28.
- Olds DL, Henderson CR, Chamberlin R, Tatelbaum R. Preventing child abuse and neglect: A randomized trial of nurse home visitation. Pediatrics, 1986;78:65-78.
- Olds DL, Henderson CR, Tatelbaum R, Chamberlin R. Improving the life-course development of socially disadvantages mothers: A randomized trial of nurse home visitation. American Journal of Public Health, 1988;78:1436-1435.
- Osgood DW, Johnston L, O'Malley P, Bachman J. The generality of deviance in late adolescence and early adulthood. American Sociological Review, 1988;53:81-93.
- Olds DL, Henderson C, Phelps C, et al. Effects of prenatal and infancy nurse home visitation on government spending. Medical Care, 1993;31:155-174.
- Olds DL, Henderson CR, Tatelbaum R. Intellectual impairment in children of women who smoke cigarettes during pregnancy. Pediatrics, 1994;93:221-227.
- Olds DL, Henderson CR, Tatelbaum R. Prevention of intellectual impairment in children of women who smoke cigarettes during pregnancy. Pediatrics, 1994;93:228-233.
- Olds DL, Henderson CR, Kitzman H. Does prenatal and infancy nurse home visitation have enduring effects on qualities of parental caregiving and child health at 25 to 50 months of life? Pediatrics, 1994;98:89-98.
- Olds DL, Henderson CR, Kitzman H, Cole R. Effects of prenatal and infancy nurse home visitation on surveillance of child maltreatment. Pediatrics, 1995;95:365-372.
- Olds DL, Kitzman H, Cole R, Robinson J. Theoretical foundations of a program of home visitation for pregnant women and parents of young children. Journal of Community Psychology, 1997;25:9-25.

- Karoly LA, et al. Benefits and costs of early-childhood interventions - A documented briefing. Santa Monica, the Rand Corporation, 1997.
-Olds DL, Eckenrode J, Henderson CR, et al. Long-term effects of home visitation on maternal life-course and child abuse and neglect: 15-year follow-up of a randomized trial. JAMA, 1997;278:637-43.
- Olds DL, Pettitt LM, Robinson J, Henderson C Jr., Eckenrode J, Kitzman H, Cole B, Powers J. Reducing risks for antisocial behaviour with a program of prenatal and early childhood home visitation. Journal of Community Psychology, 1998;26(1):65-84.
- De Bellis MD, et.al. Developmental traumatology, Part I: Biological Stress Systems, Biological Psychiatry, 1999;45:1259-1270.
- De Bellis MD, et al. Developmental traumatology, Part II: Brain Development Biological Psychiatry 1999;45:1270-1284.
- Olds DL, et al. Blueprints for Violence Prevention, Nurse-Family Partnership. Center for the study and Prevention of Violence, Boulder, Colorado, United States of America, 2001.
-Bakker I, Blokland G, et al. Samendelen. Methodiek voor opvoedingsondersteuning in groepen. Utrecht, NIZW, 2001.
- Mihalic S, et al. Blueprints for Violence Prevention Replications: Factors for implementation success, Center for the study and Prevention of Violence, Boulder, Colorado, United States of America, 2002.
- Olds DL. Prenatal and infancy home visiting by nurses: from randomzed trials to community replication Prevention Science, 2002;3:153-172.
- Prinsen B, Verhegge K, ten Thije I. Met ouders samen. Opvoedingsondersteuning in de preventieve zorg in Nederland en Vlaanderen, NIZW Uitgeverij, Utrecht, 2002.
- Hermanns J. Het bestrijden van kindermishandeling: welke aanpak werkt?. Woerden, CO ACT Consult, 2003.
- Ravensbergen J, et al. In zicht. Nieuwe wegen voor implementatie, van Gorcum, Assen, 2003.
- RAAK Manifest: Kindermishandeling, een zaak van volwassenen, 2004.
- Baartman H, Bullens R, Willems J. Kindermishandeling: de politiek een zorg, Amsterdam, SWP, 2005.
-Graaf I de, Riper H (2006) Zorg voor moeder en kind, programma-evaluatie VoorZorg: huisbezoeken door een verpleegkundige tijdens de zwangerschap en eerste twee levensjaren van het kind. Trimbos instituut, 2006.
